# Prediction model for psychological disorders in ankylosing spondylitis patients based on multi-label classification

**DOI:** 10.3389/fpubh.2025.1497955

**Published:** 2025-03-04

**Authors:** Kun Yang, Yifan Gong, Xiaohan Xu, Tiantian Sun, Xinning Qu, Xiaxiu He, Hongxiao Liu

**Affiliations:** ^1^Guang’anmen Hospital, China Academy of Chinese Medical Sciences, Beijing, China; ^2^Graduate School of Beijing University of Chinese Medicine, Beijing, China

**Keywords:** ankylosing spondylitis, psychological disorder, prediction model, multi-label classification, association rules

## Abstract

**Objective:**

This study aims to develop a predictive model to assess the likelihood of psychological disorders in patients with ankylosing spondylitis (AS) and to explore the relationships between different factors and psychological disorders.

**Methods:**

Patients were randomly divided into training and test sets in an 8:2 ratio. The Boruta algorithm was applied to select predictive factors, and a multi-label classification learning algorithm based on association rules (AR) was developed. Models were constructed using Random Forest (RF), K-Nearest Neighbor (KNN), RF-AR, and KNN-AR, and their performance was assessed through receiver operating characteristic (ROC) curves on the test set.

**Results:**

A total of 513 AS patients were included, with 410 in the training set and 103 in the test set. The Boruta algorithm identified five key variables for the model: fatigue, ASAS-HI score, disease duration, disease activity, and BMI. The RF-AR model performed best, with an accuracy of 0.89 ± 0.06, recall of 0.78 ± 0.1, F1-score of 0.86 ± 0.08, Hamming loss of 0.05 ± 0.03, and a Jaccard similarity coefficient of 0.75 ± 0.12. The area under the curve (AUC) for the training set was 0.94.

**Conclusion:**

This study developed a predictive model for assessing the risk of psychological disorders in AS patients. The model effectively captures the presence of psychological disorders, providing clinicians with valuable insights for adjusting treatment strategies.

## 1 Introduction

Ankylosing spondylitis (AS) is a chronic, progressive autoimmune disease characterized by persistent pain and restricted mobility, which can severely affect patients’ mental health (1). Research consistently shows that AS patients are at a significantly higher risk of psychological disorders compared to the general population, particularly depression and anxiety. According to the results from the International Map of Axial Spondyloarthritis, 59.4% of AS patients report poor mental health status (2). A meta-analysis involving 16 studies and 4,753 AS patients found that 38% of AS patients experienced depressive symptoms, with 15% suffering from moderate-to-severe depression (3). Similarly, anxiety was prevalent in 38% of AS patients (4). The presence of psychological disorders such as depression and anxiety not only exacerbates the disease activity of AS but also further diminishes the patients’ quality of life (5, 6). Moreover, these mental health issues may negatively affect treatment outcomes, hindering the overall management of the disease (7).

Despite the high incidence of psychological disorders in AS patients (2, 8), early detection and intervention remain significant challenges. Currently, there are no specific prediction models for psychological disorders in AS patients. In the broader context of autoimmune diseases, several single-label models have been developed to predict mental health conditions. Tennenhouse LG used logistic regression, neural network and random forest methods to predict depression and anxiety, respectively, in individuals with immune-mediated inflammatory diseases (9). YANG predicts the risk of depression in connective tissue patients through six machine learning models including SVM and RFC (10). However, these models rely on single-label classification, which predicts only one condition at a time and does not consider the co-occurrence or relationships between multiple psychological disorders. While they offer insights into individual psychological conditions, they fail to capture the complexity of co-morbid mental health issues, which are common in AS.

Clinically, patients with depression often exhibit anxiety symptoms, and vice versa (11). Depression and anxiety are often external signs of underlying stress. Our research showed that more than 50% of AS patients with psychological disorders experienced two or more conditions simultaneously. Relying solely on single-label models to predict one condition at a time can lead to an incomplete understanding of a patient’s mental health status, ignoring the intricate relationships between different mental conditions. This finding highlights the need to use multi-label classification in predicting mental health outcomes. Predicting only a single psychological disorder overlooks co-occurrence patterns and the relationships between disorders. This oversight underestimates the risk of patients having multiple concurrent disorders, limiting the predictive power of these models.

To overcome these limitations, this study aims to develop a multi-label classification model to predict psychological disorders in AS patients. This model identifies depression, anxiety, and stress, while also accounting for the correlations between them, thereby improving predictive accuracy and reliability. By using multi-label classification, we aim to capture co-occurrence patterns of psychological disorders more comprehensively, providing strong support for clinical interventions and personalized patient care.

## 2 Materials and methods

### 2.1 Study population

Data for this study were sourced from the CERTAIN database, a specialized registry for ankylosing spondylitis (AS) within traditional Chinese medicine rheumatology. Data collection spanned from July 2022 to July 2024. The CERTAIN database comprises data from AS patients at seven hospitals across China: Guang’anmen Hospital, Southwest Hospital, the Affiliated Hospital of Liaoning University of Traditional Chinese Medicine, Shanghai Guanghua Integrated Hospital, Xiyuan Hospital, Tangshan Workers’ Hospital, and the Affiliated Hospital of Shandong University of Traditional Chinese Medicine. Inclusion criteria included: (1) Patients meeting the modified 1984 New York criteria for AS diagnosis or the 2009 ASAS criteria for axial spondyloarthritis (SpA); and (2) Patients who voluntarily participated in clinical assessments and provided informed consent. Patients unwilling to participate or with incomplete assessment data were excluded. Baseline outpatient data from AS patients recorded in the CERTAIN database between July 2022 and July 2024 were selected for this study. The study was approved by the Ethics Committee of Guang’anmen Hospital, China Academy of Chinese Medical Sciences (Approval No. 2022-108-KY). Informed consent was obtained from all participants. The clinical trial registration number is ChiCTR2200058934.

### 2.2 Classification and diagnostic criteria for psychological disorders

Psychological status was assessed using the Depression Anxiety Stress Scales-21 (DASS-21), a widely used tool for measuring levels of depression, anxiety, and stress over the past week. The DASS-21 comprises 21 items divided into three subscales: Depression, Anxiety, and Stress, each with seven items. Each item is rated on a scale of 0 (“Did not apply to me at all”) to 3 (“Applied to me most of the time”). Subscale scores are multiplied by 2 to obtain the final score. For the Depression subscale, scores from 0 to 4 are considered normal. In the Anxiety subscale, 0 to 3 is normal, and in the Stress subscale, 0 to 7 is within the normal range. Scores exceeding these thresholds suggest the presence of the respective psychological disorder.

### 2.3 Data collection

This study gathered a range of patient data, including demographic details (age, gender, BMI), medical history (hypertension, diabetes), smoking history, disease duration, comorbidities (uveitis, IBD), and family history. Disease activity was measured using the Bath Ankylosing Spondylitis Disease Activity Index (BASDAI) and the Ankylosing Spondylitis Disease Activity Score (ASDAS-CRP), which is based on C-reactive protein. Spinal function was assessed using the Bath Ankylosing Spondylitis Functional Index (BASFI). Additionally, patients’ global assessment (PGA), nighttime low back pain VAS scores, chronic disease-related fatigue (FACIT-F), Ankylosing Spondylitis Health Index (ASAS-HI), and hematological indicators (C-reactive protein, HLA-B27) were collected and analyzed. Based on WHO age classification, patients were divided into three age groups: ≤44 years, 45–59 years, and ≥ 60 years. According to WHO’s BMI classification, patients were grouped into <18.5 kg/m^2^, 18.5–23.9 kg/m^2^, and ≥ 25 kg/m^2^ categories. After statistical analysis of disease duration, patients were divided into four groups based on quartiles: ≤5 years, 5–10 years, 10–18 years, and > 18 years. CRP levels were categorized as normal (<10 mg/L) or abnormal (≥10 mg/L) based on a threshold of 10 mg/L.

BASDAI (12) was used to assess AS disease activity. It consists of six questions addressing five key symptoms of AS: fatigue, spinal pain, peripheral joint pain/swelling, enthesitis, and morning stiffness (severity and duration). The average score of these five symptoms over the past week is used to compute the BASDAI score (range 0–10), where higher scores reflect greater disease activity. A BASDAI score < 4 is classified as inactive disease, while ≥4 indicates active disease.

ASDAS-CRP (13) combines self-reported measures and inflammatory markers (CRP) to evaluate AS disease activity. It includes BASDAI’s spinal pain, peripheral joint pain/swelling, and morning stiffness duration. CRP is measured in mg/dL. The evaluation criteria used in this study classify disease activity into four categories. A score greater than 3.5 indicates very high disease activity, scores between 2.1 and 3.5 reflect high disease activity, values ranging from 1.3 to 2.1 represent moderate disease activity, and scores below 1.3 indicate inactive disease.

BASFI (14) was used to assess the functional status of AS patients. It includes 10 questions related to daily activities and tasks. Each question is rated on a 10 cm horizontal scale, from 0 (easy) to 10 (impossible). Higher average scores indicate more severe functional impairment. For BASFI, patients were divided into two groups based on the median score, with scores of 1 or below categorized in one group, and scores greater than 1 in the other.

The FACIT-Fatigue Scale (15) was used to evaluate patient fatigue. It contains 13 items, and the total score of all items represents the fatigue index, with higher scores indicating less fatigue. Fatigue levels were categorized into two groups, with scores ranging from 40 to 52 indicating little or no fatigue, and scores from 0 to 39 indicating significant fatigue.

The Ankylosing Spondylitis Health Index (ASAS-HI) is a comprehensive tool for assessing the health status of AS patients (16). It consists of 17 items covering various aspects such as pain, work ability, sleep quality, emotional status, social interaction difficulties, and mobility restrictions. Each problem scores 1 point, while the absence of problems scores 0. Higher scores indicate a greater impact on patient health. ASAS-HI scores were divided into three levels in this study. Patients with scores of 5 or below were categorized as having good overall health, those with scores between 6 and 11 were classified as moderate, and those with scores of 12 or higher were considered to have poor overall health.

A visual analog scale (VAS) is used, with scores ranging from 0 to 10, where higher scores indicate worse overall patient evaluation. PGA was classified into two groups. Patients with scores of 5 or below were grouped into one category, while those with scores above 5 were placed in another.

Nighttime low back pain was assessed using the VAS method, with scores ranging from 0 to 10. Higher scores indicate more severe symptoms. Those with scores of 1 or below formed one group, while those with scores greater than 1 formed the other.

### 2.4 Data analysis

Frequencies, percentages, means, and standard deviations (SD) were used to describe the cohort’s sociodemographic and disease characteristics. Additionally, the prevalence of depression, anxiety, and stress, assessed via the DASS-21 scale, was reported for the study population.

### 2.5 Feature selection and importance ranking

In this study, nineteen features were analyzed using the Boruta package to extract and rank key variables. The Boruta algorithm is an all-relevant feature selection method that iterates multiple times to assess the importance of each feature by comparing it with shadow features, which are randomly permuted copies of the original features. The importance of each feature is calculated based on its Z-score in a random forest classifier. During each iteration, the importance of the original features is compared to the maximum importance of the shadow features, which serve as a baseline. If a feature’s importance exceeds that of the shadow features, it is considered significant. This iterative process is repeated until the features stabilize or a pre-defined number of iterations is reached.

The Boruta algorithm operates by first generating a shadow feature set, where each feature is randomly shuffled to ensure that these shadow features hold no predictive power. Then, a random forest classifier is trained on the dataset, and the importance of each feature is determined by evaluating its contribution to the overall model performance. After the features are evaluated, those with higher importance than at least one shadow feature are marked as “green,” indicating their relevance for model construction. Features with uncertain significance are marked as “yellow,” and those deemed irrelevant are marked as “red.” Only the “green” features were retained for further analysis and model building, ensuring that only the most relevant features were considered in the subsequent stages of the study.

### 2.6 Model construction

#### 2.6.1 Basic model construction

In this study, the modeling process was carried out using the sci-kit-learn library (version 0.19.2) in Python (version 3.7.1). The dataset was divided into a training set (410 cases, 80%) and a test set (103 cases, 20%). Model building and hyperparameter tuning were conducted on the training set, with final performance evaluated on the test set. Model reliability was assessed using 10-fold cross-validation and external test set validation.

The Random Forest (RF) algorithm first used bootstrap sampling with replacement to generate N new datasets from the original data. The Gini coefficient was used to evaluate the decision tree splitting points. The model was tested using out-of-bag data to estimate the error rate.

The K-Nearest Neighbor (KNN) algorithm first normalized the data to ensure equal weighting for all features during distance calculations. The optimal value of K was determined through cross-validation. For each sample, the Euclidean distance to all training samples was calculated, and the K nearest neighbors were selected based on the shortest distance.

#### 2.6.2 Model construction based on association rules

To explore the underlying relationships among variables in the dataset, association rules were employed. These rules are commonly expressed as “A → B,” meaning that if A occurs, B is likely to follow. The Apriori algorithm was chosen for rule generation due to its suitability for smaller datasets. The Apriori algorithm works by first scanning the dataset to identify frequent itemsets—combinations of items that appear together with sufficient frequency. Once the frequent itemsets are identified, potential association rules are generated. These rules are evaluated based on three key metrics: support, confidence, and lift. Support refers to the frequency with which the itemset appears in the dataset, confidence measures the likelihood that B occurs given A, and lift indicates the strength of the rule, compared to random chance.

Once the candidate rules are generated, they are filtered based on predefined thresholds for support, confidence, and lift. The rules that meet these criteria are selected as valid association rules that reveal the relationships between different features in the dataset. These selected rules are then integrated with the original features to form an extended feature set, which includes both the original variables and the newly discovered relationships. This extended feature set was subsequently used to build two multi-label classification models: a multi-label Random Forest model based on association rules (RF-AR) and a multi-label KNN model based on association rules (KNN-AR). By incorporating association rules into the feature set, these models are better equipped to predict multiple psychological disorders simultaneously, accounting for the interdependencies among different conditions in AS patients.

### 2.7 Model evaluation

**Figure 1:**
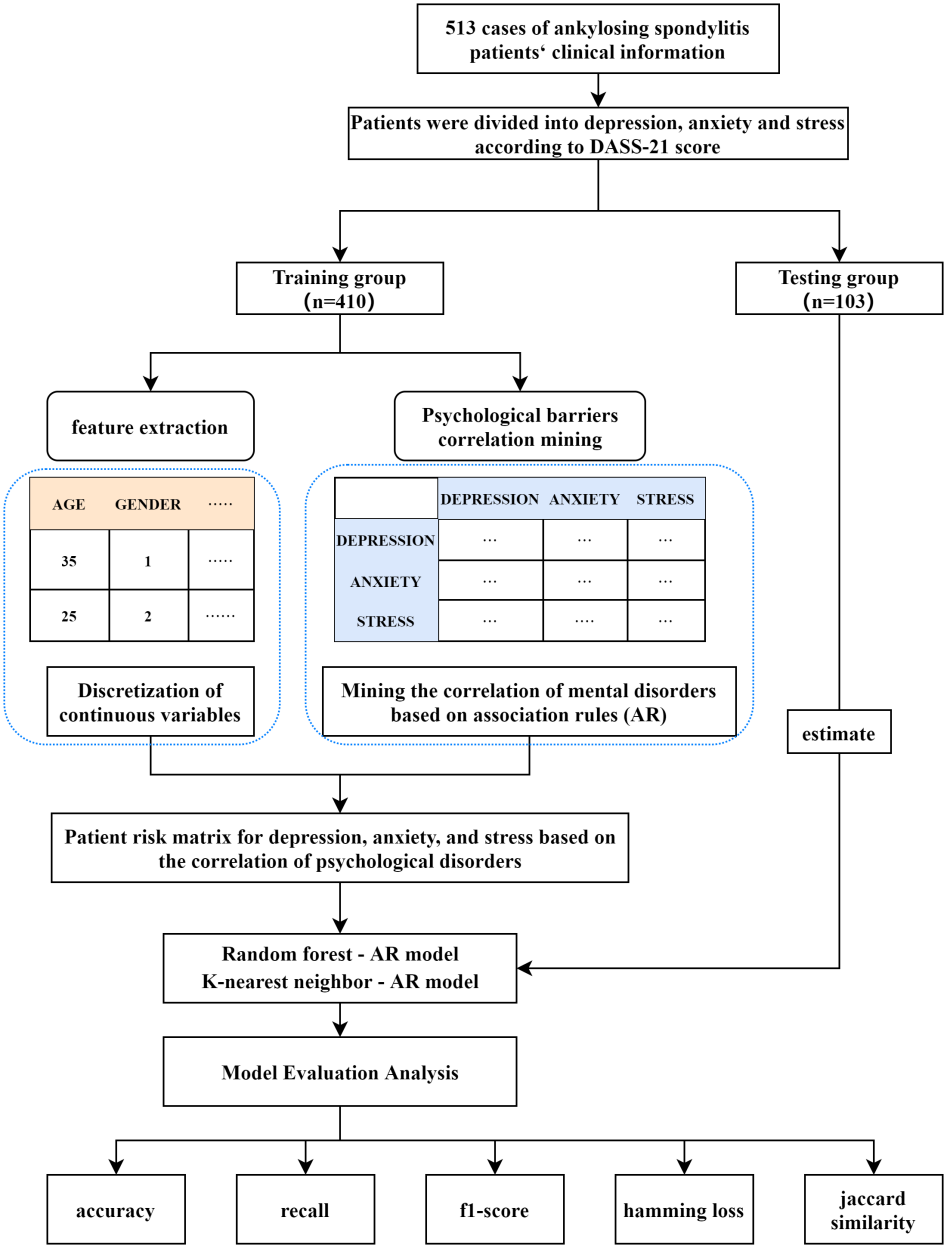
Model framework diagram. DASS-21, Depression Anxiety and Stress Scale; AR, association rules.

In multi-label classification tasks, evaluating model performance typically involves several metrics to comprehensively assess how the model performs for each label and overall. In addition to common metrics such as accuracy, recall, and F1-score, two other important measures—Jaccard similarity coefficient and Hamming loss—are frequently used to assess the performance of multi-label models. The research framework is shown in Figure 1.

The Jaccard similarity coefficient evaluates both the precision and recall of predicted labels, measuring the similarity between predicted and actual label sets:


$$J\left(P,N\right)=\frac{\left|P\cap N\right|}{\left|P\cup N\right|}$$

Where P represents the set of predicted labels, and N represents the set of true labels.

Hamming Loss, on the other hand, measures the difference between the predicted and true labels:


$$HL=\frac{1}{M}\frac{1}{C}\overset{M}{\underset{i=1}{\sum }}Li$$

$$Li=\frac{1}{C}\left(\left|\mathrm{Pi}|+|Ni|-2|\mathrm{Pi}\cap Ni\right|\right)$$

Where Pi and Ni represent the predicted and true label sets for the i-th sample, and C is the total number of labels, used to normalize the Hamming loss.

## 3 Result

### 3.1 Patient baseline characteristics

A total of 513 patients with ankylosing spondylitis (AS) were included in this study. The average age of the patients was 38.96 ± 11.40 years, with 70.96% of the patients being under 44 years old, falling within the young and middle-aged category as defined by the WHO. Of the total patients, 396 were male, accounting for 77.19%. Further patient details are presented in Table 1.

**Table 1:** Characteristics of the study cohort (*n* = 513).

Variables	Total (*n* = 513)
Age, Mean ± SD	38.96 ± 11.40
≤44 years	364 (70.96)
45–59 years	121 (23.59)
≥60 years	28 (5.46)
Gender, n(%)	
Male	396 (77.19)
Female	117 (22.81)
BMI, Mean ± SD	25.61 ± 8.29
<18.5 kg/m^2^	26 (5.07)
18.5–23.9 kg/m2	208 (40.55)
≥24 kg/m2	279 (54.39)
Disease Duration, Mean ± SD	12.77 ± 11.06
≤5	140 (27.29)
5<disease duration≤10	128 (24.95)
10<disease duration≤18	92 (17.93)
>18	153 (29.82)
Family History, yes, n(%)	128 (24.95)
Smoke, yes, n(%)	168 (32.75)
Comorbid conditions	
Hypertension, yes, n(%)	33 (6.43)
Diabetes, yes, n(%)	15 (2.92)
Extra articular manifestation	
Uveitis, yes, n(%)	97 (18.91)
IBD, yes, n(%)	34 (6.63)
HLA-B27, yes, n(%)	317 (61.79)
ASDAS-CRP, Mean ± SD	2.09 ± 1.03
<1.3	106 (20.66)
1.3 ≤ ASDAS-CRP<2.1	170 (33.14)
2.1 ≤ ASDAS-CRP ≤ 3.5	191 (37.23)
>3.5	46 (8.97)
BASDAI, Mean ± SD	2.60 ± 2.17
<4	409 (79.72)
≥4	104 (20.27)
BASFI, Mean ± SD	2.00 ± 2.52
≤1	278 (54.19)
>1	235 (45.81)
FACIT-F, Mean ± SD	38.35 ± 9.04
Little or no fatigue	297 (57.89)
Significant fatigue	216 (42.11)
ASAS-HI, Mean ± SD	6.38 ± 5.04
≤5	264 (51.46)
6–11	174 (33.92)
≥12	75 (14.62)
PGA, Mean ± SD	4.36 ± 2.68
≤5	327 (63.74)
>5	186 (36.26)
Pain vas, Mean ± SD	1.95 ± 2.38
≤1	272 (53.02)
>1	241 (46.98)
CRP, Mean ± SD	10.16 ± 15.19
≤10 mg/L	367 (71.54)
>10 mg/L	146 (28.46)

BMI, Body Mass Index; IBD, Inflammatory Bowel Disease; BASDAI, Bath Ankylosing Spondylitis Disease Activity Index; BASFI, Bath Ankylosing Spondylitis Functional Index; ASAS-HI, Assessments of Spondyloarthritis international society Health Index; FACIT-F, Functional Assessment of Chronic Illness Therapy – Fatigue; PGA, patient global assessment; CRP, C-Reactive Protein.

### 3.2 Psychological disorders in patients

Among the 513 AS patients included in the study, 154 were found to have at least one psychological disorder. Of the patients with a single psychological disorder, anxiety was the most common, affecting 44 individuals (7.99%). Additionally, 79 patients (51.3% of those with psychological disorders) had two or more concurrent psychological disorders. Among these, the coexistence of all three disorders was the most prevalent, affecting 36 individuals (7.02%) (Table 2).

**Table 2:** Statistical characteristics of psychological disorders of patients in the dataset.

	No. of people (n, %)	Male (%)	Age (years)
Psychological disorders	154 (30.02)	75.97	39.19 ± 11.22
Depression only	27 (5.26%)	23 (85.19%)	41.80 ± 4.66
Anxiety only	41 (7.99%)	30 (73.17%)	36.60 ± 14.15
Stress only	7 (1.36%)	6 (85.71%)	34.40 ± 9.94
Combined psychological disorder	79 (15.40%)	23 (63.89%)	43.33 ± 17.88
Depression and Anxiety	30 (5.85%)	24 (80.00%)	46.80 ± 14.25
Anxiety and Stress	8 (1.56%)	7 (87.50%)	42.63 ± 13.29
Depression and Stress	5 (0.97%)	4 (80.00%)	42.20 ± 7.19
Depression and Anxiety and Stress	36 (7.02%)	23 (63.89%)	43.33 ± 17.88

### 3.3 Correlation analysis of psychological disorders

**Figure 2:**
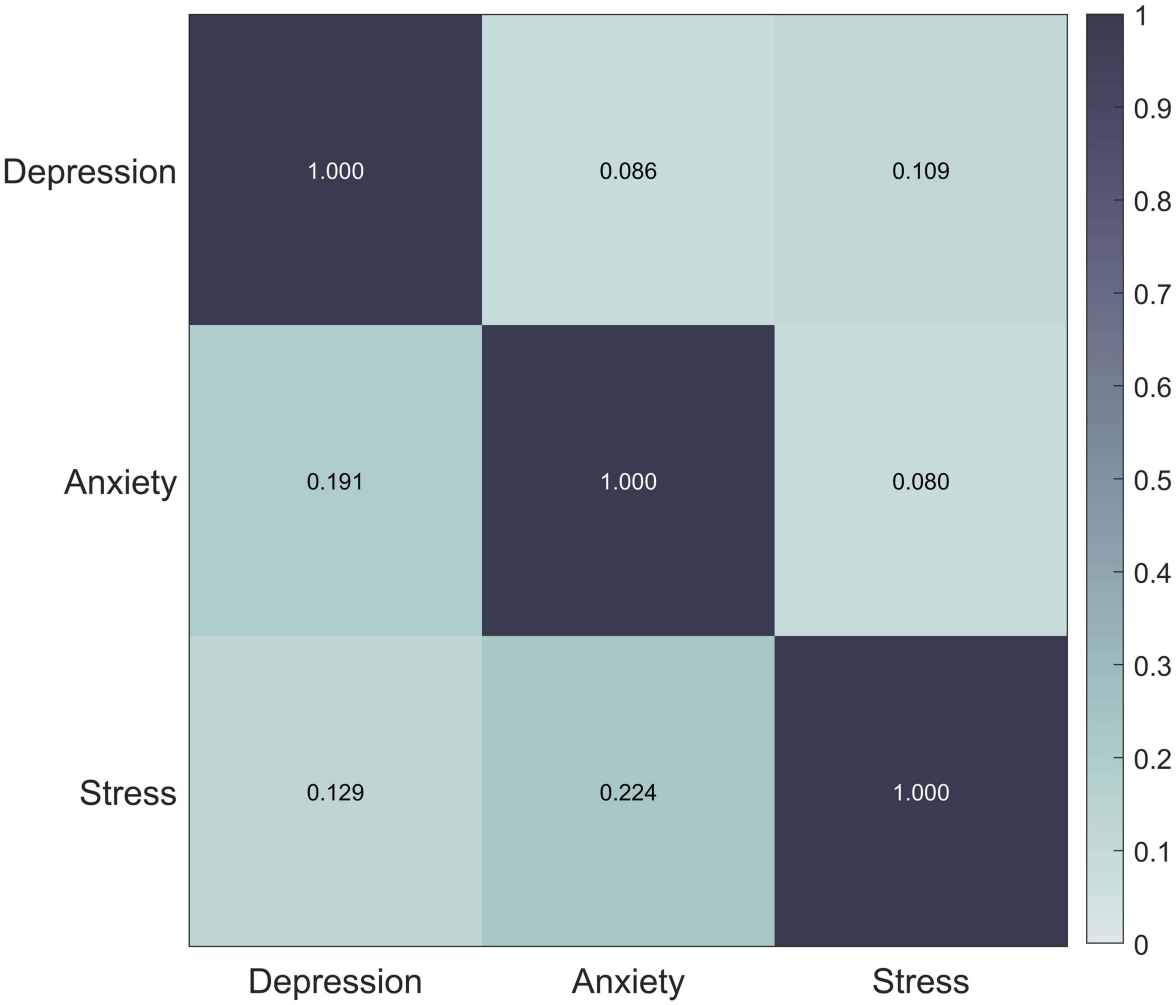
Disease correlation mining results. Correlation matrix between diseases showing the relationship between depression, anxiety and stress. Color shades indicate the strength of the correlation and the color bar on the right side represents the range of the correlation coefficients.

Association rule mining was used to analyze the correlations between psychological disorders. The minimum support (minsupp) and minimum confidence (minconf) were set to 0.01, resulting in the identification of nine association rules. Confidence was used as the correlation coefficient between psychological conditions. The correlation coefficients for the three psychological states are illustrated in Figure 2. The three pairs of psychological disorders with the highest correlation were: stress → anxiety (correlation coefficient 0.224), anxiety → depression (correlation coefficient 0.191), and stress → depression (correlation coefficient 0.129).

### 3.4 Parameter selection for RF and KNN models

**Figure 3:**
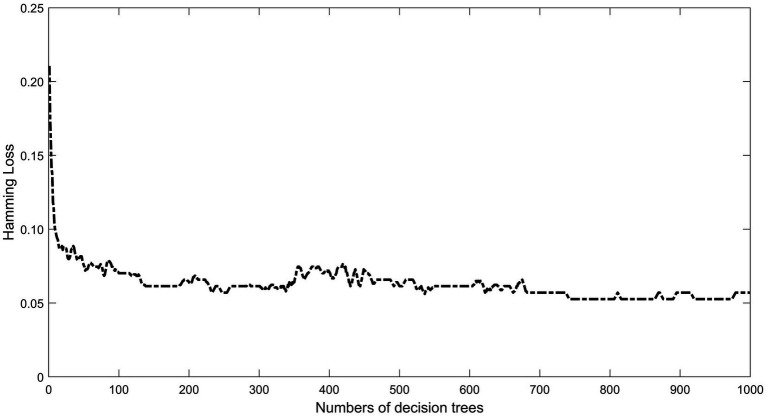
Optimal number of RF models. The X-axis represents the number of decision trees and the Y-axis represents the Hamming loss. With the increase of the number of decision trees, the Hamming loss fluctuation decreases and tends to be stable, and the model gradually tends to be stable after 54 trees, choosing tree = 100 to establish the random forest model.

In the RF model, an initial forest of 1,000 decision trees was used as the default. The “which.min” function was applied to calculate the optimal number of decision trees. The result indicated that a model with ntree = 100 produced the lowest error rate (5.6%) (Figure 3).

**Figure 4:**
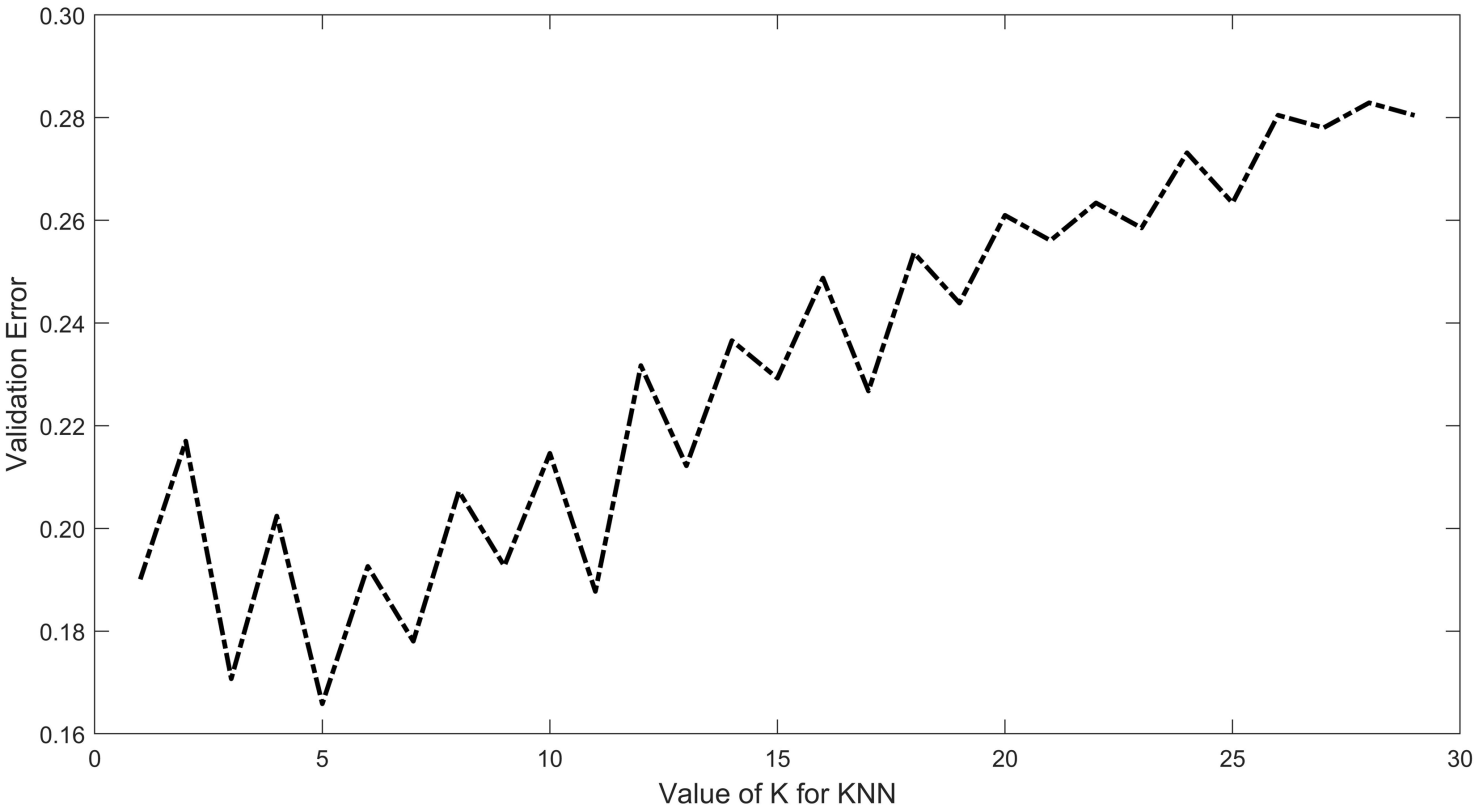
KNN model for optimal K value. The X-axis indicates the range of K values (from 1 to 30) and the Y-axis indicates the validation error. The results show that the validation error shows a fluctuating upward trend as the value of K increases, and the error is minimized at *K* = 5.

For the KNN model, cross-validation was employed to select the optimal K value. The results showed that as the value of K increased, the validation error generally rose, with a more pronounced increase after a specific threshold. The validation error exhibited some fluctuations rather than a monotonic increase. The lowest validation error was observed at K = 5 (Figure 4).

### 3.5 Feature selection and importance ranking

**Figure 5:**
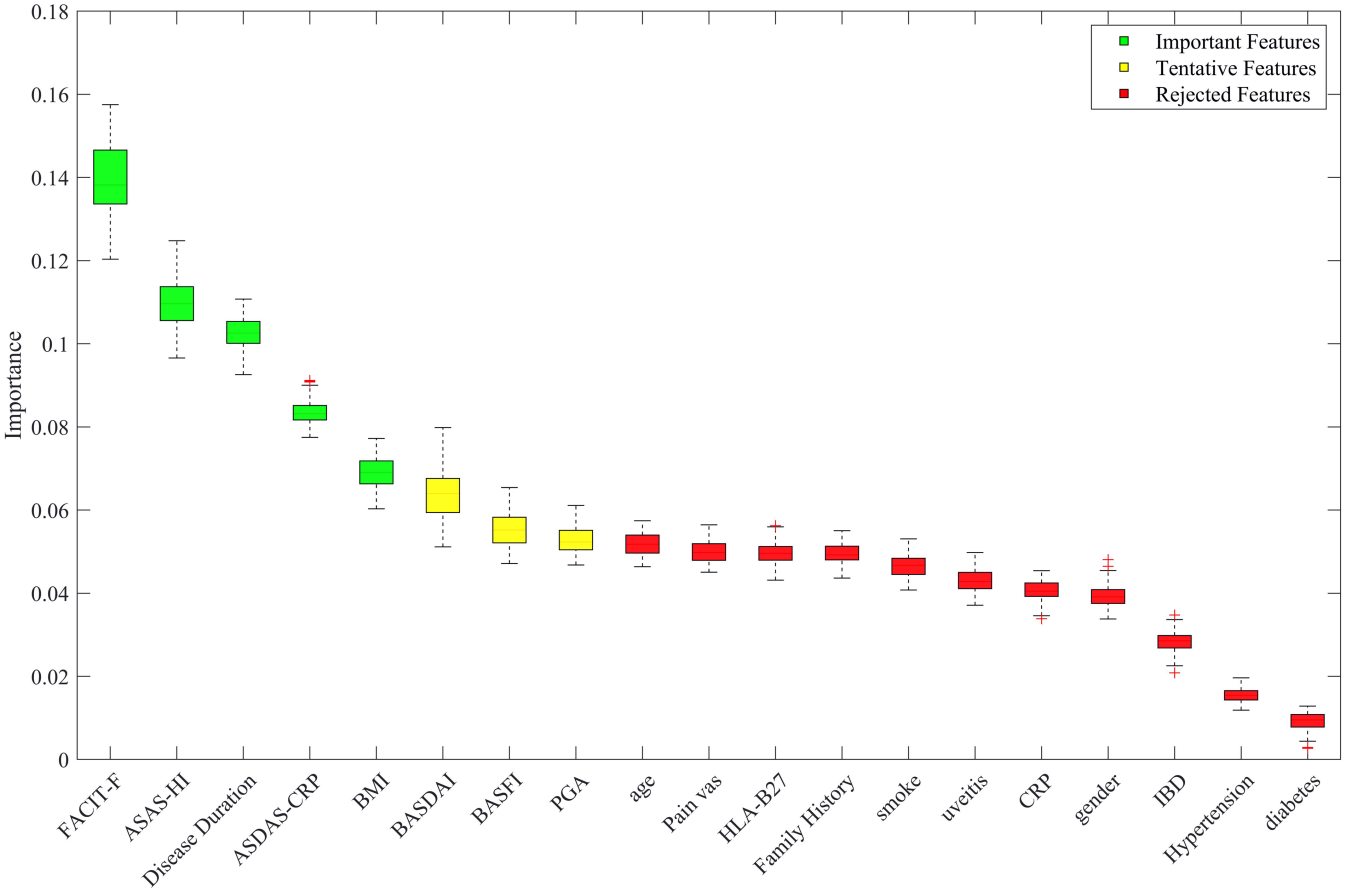
Visual ranking of importance of model variables by Boruta’s algorithm. The horizontal axis represents each candidate feature and the vertical axis represents the importance of the feature. Green boxes indicate variables identified as important features, yellow boxes indicate tentative features, and red boxes indicate rejected features.

After multiple iterations of the “Boruta” algorithm, the evaluation of each feature revealed that severe fatigue, moderate or poor ASAS health index scores, prolonged disease duration, high disease activity, and elevated BMI were the most significant predictors. Clinical features such as BASDAI, BASFI, and PGA showed lower predictive performance, and the other features were deemed irrelevant. Refer to Figure 5. For the model construction, the five key predictors selected were fatigue severity, ASAS health index, disease duration, disease activity, and BMI.

### 3.6 Comparison of model performance before and after incorporating association rules

A multi-label dataset was constructed using patient information, with anxiety, depression, and stress, both individually and in combination, as the label set. The dataset was divided into training and testing sets, with 80% of the samples used for training and 20% for testing, and ten-fold cross-validation was performed. Table 3 demonstrates that incorporating association rules significantly improved the accuracy of the model in predicting patients’ psychological states. The results show a marked increase in the Jaccard index after adding association rules, indicating a higher consistency between the model’s predictions and the actual results within the label set intersections, which reflects better multi-label prediction performance. The Jaccard similarity coefficient is a metric that measures the proportion of shared labels between the predicted and true label sets, thus indicating how well the model is capturing the relationships between different psychological disorders. A higher Jaccard index indicates better model performance in handling multiple labels simultaneously, which is crucial for multi-label classification tasks like this one.

**Table 3:** Comparison of performance across different models.

	Accuracy	Micro_recall	Micro_f1-score	Hamming_loss	Jaccard_similarity
KNN	0.67 ± 0.07	0.25 ± 0.09	0.32 ± 0.10	0.18 ± 0.05	0.17 ± 0.06
KNN-AR	0.82 ± 0.05	0.62 ± 0.12	0.75 ± 0.09	0.07 ± 0.02	0.60 ± 0.12
RF	0.68 ± 0.06	0.23 ± 0.08	0.32 ± 0.09	0.16 ± 0.04	0.16 ± 0.05
RF-AR	0.89 ± 0.06	0.78 ± 0.10	0.86 ± 0.08	0.05 ± 0.03	0.75 ± 0.12

**Figure 6:**
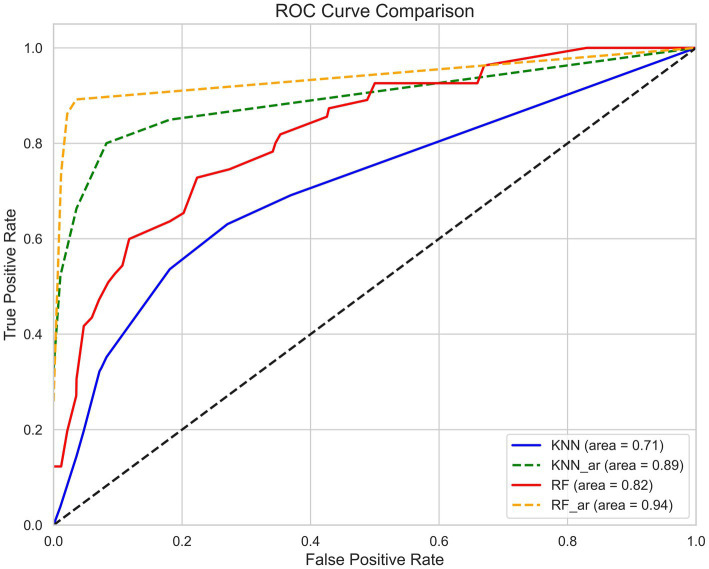
ROC curve of the AS psychological disorder prediction model. The X-axis represents the False Positive Rate and the Y-axis represents the True Positive Rate. Area Under the Curve (AUC) is used to evaluate the model performance, with AUC closer to 1 indicating better predictive performance. The graph compares the classification performance of the four models KNN (blue, AUC = 0.71), KNN-AR (green, AUC = 0.89), RF (red, AUC = 0.82) and RF-AR (yellow, AUC = 0.94). The results show that the RF_AR model has the highest AUC and has the best classification ability.

In addition to the Jaccard index, the Hamming loss was also used to evaluate the model’s performance. Hamming loss calculates the fraction of incorrectly predicted labels, where a value of 0 indicates perfect prediction and a value of 1 indicates total misclassification. Lower Hamming loss values signify that the model is better at predicting the exact set of labels for each patient. The results show that after adding association rules, there is a notable improvement in both the Jaccard index and Hamming loss, which indicates better multi-label prediction performance. Compared to the KNN-AR model, the RF-AR model exhibits significant advantages in terms of accuracy, recall, Hamming loss, and Jaccard index. The AUC for the test sets of the four models ranges from 0.71 to 0.94, with the RF-AR model achieving an AUC of 0.94, highlighting its excellent classification capability and accuracy (Figure 6).

## 4 Discussion

AS is an inflammatory disease characterized by chronic lower back pain, stiffness, and fatigue, with the potential for disability. It not only affects the physical domain but also has a significant impact on mental health. In our study, we assessed the psychological status of 513 AS patients from China using the DASS-21 scale and developed predictive models for psychological outcomes based on collected clinical data. The DASS-21 results revealed that 30.02% of patients experienced psychological disorders. Using the Boruta algorithm to visualize feature importance, we identified fatigue, ASAS-HI score, disease duration, disease activity, and BMI as key factors influencing mental health.

Fatigue results from various factors affecting local tissues or systemic organs. It leads to reduced muscle capacity, diminished work ability, and a decline in energy and motivation, ultimately impairing overall bodily function (17) describe it as persistent and systemic exhaustion. This condition reduces functional capacity and daily activity levels. It often leads to disability and is one of the most common complaints among AS patients (18). Fatigue and psychological disorders may share overlapping symptoms, such as tiredness, reduced attention, and decreased motivation, which can exacerbate each other. Fatigue can also affect psychological health indirectly by impairing physical function. Zhou’s research found that patients with fatigue tend to have poorer physical function and exhibit more pronounced anxiety (19). MRI imaging of AS patients with fatigue revealed a significant increase in left thalamus volume. Clinical data also showed that these patients experienced more severe psychological disorders (20). Thalamic changes are a prominent brain alteration in patients with depression (21). This suggests a neuropsychological link between fatigue and psychological disorders in AS patients.

The ASAS-HI consists of 17 patient-reported items covering categories such as pain, emotional function, sleep, sexual function, activity, self-care, community life, and employment. It offers comprehensive insights into the overall health of AS patients. A higher ASAS-HI score indicates poorer overall function, which in our study also suggested a higher risk of psychological disorders. Qu et al. found that when patients experienced impaired health (ASAS-HI score > 5), their psychological state was significantly more affected compared to those in better health (22).

Our study similarly emphasizes the important role of disease duration in the psychological health of AS patients. In our cohort, the average disease duration was 12.77 ± 11.06 years. Notably, 72.7% of patients had a disease duration exceeding 5 years, and 47.75% had a duration of over 10 years. Patients with a longer disease course often experience more severe psychological issues, likely due to chronic pain, diminished quality of life, and repeated unsuccessful treatments. As the disease progresses, patients may gradually lose hope in treatment, which increases the risk of depression and anxiety. Furthermore, the prolonged disease burden can lead to changes in social roles, such as reduced work capacity and lighter family responsibilities, which further intensify psychological stress. Therefore, patients with longer disease durations tend to have poorer mental health, necessitating long-term attention to their psychological needs in clinical practice.

ASDAS-CRP is the preferred indicator for assessing disease activity in ankylosing spondylitis (AS), as it combines both clinical symptoms and laboratory markers of inflammation. Our results Indicate that higher disease activity is associated with a heavier psychological burden, leading to an increased incidence of mental health disorders. This finding is consistent with a 2006 study by J. Martindale et al. (23). Patients with high disease activity often experience more frequent symptom flare-ups and a significant decline in quality of life. The uncertainty about the future and the sense of powerlessness in managing their condition substantially increase their psychological stress. Therefore, managing disease activity in AS patients not only improves their physical health but also has a positive impact on their mental well-being.

In addition, BMI was identified as a key factor influencing the psychological health of AS patients in this study. Obesity is closely associated with mental health disorders (24), and 54.39% of our study participants were classified as overweight. Excess weight can directly affect AS disease activity (25), further impacting daily life and psychological health. Overweight patients may also face self-esteem issues, social interaction difficulties, and reduced mobility due to increased joint stress, which can exacerbate mental health problems. Thus, abnormal BMI affects not only physical health but is also strongly linked to psychological well-being.

The findings of this study revealed that more than 50% of patients had two or more coexisting psychological disorders, underscoring the complexity of mental health issues in AS patients. Correlation analysis of the three psychological disorders showed the strongest association between anxiety and depression, which aligns with previous literature on the comorbidity of these conditions (26). Depression can make patients feel fatigued and demotivated, reducing their willingness to engage in treatment. Anxiety, on the other hand, may cause excessive concern about side effects or doubts about treatment effectiveness, affecting adherence to medication and medical advice. The presence of multiple psychological disorders simultaneously often compromises treatment adherence, resulting in worsened outcomes, increased disease activity, and a higher risk of complications. Since depression and anxiety often coexist and influence each other, clinical psychological evaluations in AS patients should consider a multidimensional approach to avoid missing underlying mental health issues. Early intervention targeting psychological disorders may help improve patient adherence to treatment and overall prognosis.

The multi-label Random Forest (RF) and K-Nearest Neighbors (KNN) models developed in this study significantly improved the accuracy of predicting psychological disorders compared to single-label models. Single-label models address each psychological disorder (e.g., depression, anxiety, stress) separately, which can overlook their interactions. In contrast, multi-label classification models capture these co-occurrence patterns in a single prediction, offering a more comprehensive view of the patient’s mental health. Compared to single-label models, multi-label classification models significantly enhance prediction accuracy and reliability. Co-occurring psychological disorders in AS patients significantly impact their overall health and treatment adherence. By predicting multiple psychological disorders simultaneously, multi-label models can help clinicians identify high-risk patients with multiple mental health issues. This approach not only supports more personalized psychological interventions but also aids in crafting more precise treatment plans. In this study, the RF model achieved an accuracy of 0.68 ± 0.06, a Hamming loss of 0.16 ± 0.04, and a Jaccard similarity coefficient of 0.16 ± 0.05, all significantly outperforming the KNN model.

By integrating association rules into the model, the study significantly enhanced the reliability of predictions. Association rules enable predictions by identifying frequent patterns in the data, which reduces misclassifications. By analyzing association rules, the model can predict the likelihood of additional psychological disorders based on existing data, minimizing the risk of omissions and misclassifications. The results showed that the RF-AR model achieved an accuracy of 0.89 ± 0.06, a recall rate of 0.78 ± 0.1, an F1-score of 0.86 ± 0.08, a Hamming loss of 0.05 ± 0.03, and a Jaccard similarity coefficient of 0.75 ± 0.12. These metrics demonstrate that the inclusion of association rules significantly improved the RF model’s performance, enabling it to effectively capture co-occurrence patterns between psychological disorders. Similarly, the KNN-AR model achieved an accuracy of 0.82 ± 0.05, a recall rate of 0.62 ± 0.12, a Hamming loss of 0.07 ± 0.02, and a Jaccard similarity coefficient of 0.6 ± 0.12, which were substantially higher than those of the KNN model without association rules. Both models showed a marked increase in their Jaccard similarity coefficients after incorporating association rules, suggesting that the predicted label sets became more similar to the actual label sets. This implies that the models not only correctly predicted more labels but also more accurately predicted label combinations, improving prediction accuracy and consistency. Validation on the test set showed that the RF model with association rules achieved an AUC of 0.94, significantly higher than the 0.82 achieved without association rules. This demonstrates that the introduction of association rules greatly enhanced the model’s classification performance.

This study developed a predictive model for psychological disorders in AS patients using RF, KNN, and association rule-based models. The results indicate that the RF-AR model outperformed both the standalone RF and KNN models in terms of sensitivity, specificity, and diagnostic accuracy. This model provides valuable insight into the psychological risk management of AS patients by allowing for early and effective identification of high-risk individuals.

Depression, anxiety, and stress are common and often co-exist in people with ankylosing spondylitis, which complicates treatment strategies. The multi-label classification model provides a comprehensive view of a patient’s mental health, capturing the complex relationships between disorders. This enables the design of personalized treatment plans that address the specific psychological needs of each patient. For example, patients with both depression and anxiety could benefit from a combined approach, such as cognitive-behavioral therapy (CBT) and pharmacological treatment. In contrast, those with stress-related symptoms may benefit from stress management techniques alongside traditional AS therapies. Such personalized care could improve outcomes by addressing the patient’s overall well-being, rather than just individual symptoms.

Early intervention is crucial for managing psychological disorders in AS patients. Predicting depression or anxiety early, before they significantly impact quality of life or disease progression, allows for preemptive care. Early interventions, including counseling, stress reduction, and psychological support, may prevent the worsening of these disorders and improve long-term prognosis. By integrating psychological assessments into routine AS care, clinicians can manage both physical and mental health proactively, leading to more effective disease management.

While the model demonstrated significant promise, there are several limitations that need to be addressed. The model’s performance was based solely on data from the current cohort, and its applicability to other populations remains uncertain. Future studies should validate the model using datasets from diverse clinical settings to assess its robustness and broader applicability. Another limitation is the relatively small sample size. Although ten-fold cross-validation was used to enhance reliability, a larger sample would yield more robust results and improve generalizability. Future research should also consider integrating real-time data collection tools, such as wearable devices or mobile health apps, to monitor the psychological well-being of AS patients continuously. Finally, integrating this predictive model into clinical decision support systems, such as electronic health records (EHRs), could alert healthcare providers to patients at risk of psychological disorders, facilitating earlier intervention and personalized care.

## 5 Conclusion

The RF-AR model developed in this study effectively predicts psychological disorders in patients with AS. Key clinical predictors include fatigue, ASAS-HI score, disease duration, disease activity, and BMI. This model supports early identification of high-risk patients and the development of personalized treatment plans, demonstrating the broad potential of combining multi-label classification with association rules in mental health assessments. Future research should focus on further validating and optimizing the model, providing more effective tools for the comprehensive management of AS patients.

## Data Availability

The raw data supporting the conclusions of this article will be made available by the authors, without undue reservation.
